# Unique Association of Rare Cardiovascular Disease in an Athlete With Ventricular Arrhythmias

**Published:** 2014-09-01

**Authors:** V. Santomauro, M. Contursi, S. Dellegrottaglie, G. Borsellino

**Affiliations:** 1 Sports Cardiology Center, “Check-Up Day-Surgery” Centro Polispecialistico, Salerno, Italy; 2 Division of Cardiology, Ospedale Medico-Chirurgico “Villa dei Fiori”, Acerra, Napoli, Italy and Z. and M.A. Wiener Cardiovascular Institute and H.R. Kravis Center for Cardiovascular Health, Mount Sinai Medical Center, New York, NY, USA; 3 Radiology Section, “Check-Up Day-Surgery” Centro Polispecialistico, Salerno, Italy

**Keywords:** left-dominant arrhythmogenic cardiomyopathy, Kommerell’s diverticulum, ventricular arrhythmias, cardiac magnetic resonance, sports elegibility

## Abstract

Ventricular arrhythmias are a leading cause of non-elegibility to competitive sport. The failure to detect a significant organic substrate in the initial stage of screening does not preclude the identification of structural pathologies in the follow-up by using advanced imaging techniques.

Here we report the case of a senior athlete judged not elegible because an arrhythmia with the morphology consistent with the origin of the left ventricle, in which subsequent execution of a cardiac MR and a thoracic CT scan has allowed the identification of an unique association between an area of myocardial damage, probable site of origine of the arrhythma, and a rare aortic malformation.

## Introduction

Ventricular arrhythmias are a leading cause of non-elegibility to competitive sport. [1] As in the non-athletic population the presence of an anomaly of the myocardium is a major determinant of poor prognosis of the arrhythmias. [2] However, in the absence of the stuctural heart disease also frequent and complex ventricular arrhythmias are associated with good prognosis [3] and do not usually controindicate sports activity. [4] Therefore, a thorough diagnostic work with non-invasive tests and sometimes invasive is required to rule out heart disease. The morphology of the QRS complexes on the electrocardiogram is an excellent tool to identify the site of origin of the rhythm and guide the diagnostic process. [5] The latter can now take advantage of more sophisticated imaging techniques such as magnetic resonance (MRI) and computed tomography (CT).

## Case description

**Fig. 1.**
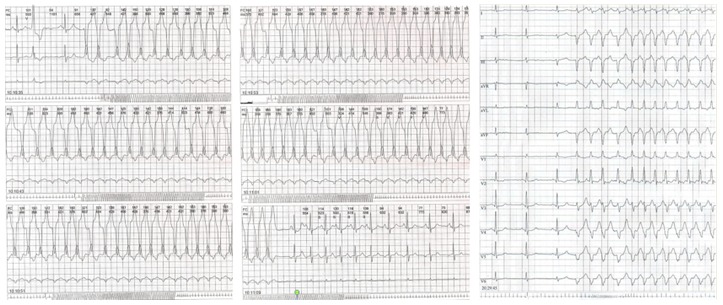
**24-hour ECG Holter monitoring**. *On the left and at the center*. Standard three-channels recording. Sustained monomorphic ventricular tachicardia, lasting about 35 seconds. *Right.* Twelve-leads option recording. Beginning of a non-sustained ventricular tachicardia with left axis deviation and right bundle branch block morphology.

A 60-year-old male high-level tennisplayer was referred at our outpatient sports cardiology clinic for palpitations after competition. 24-hour Holter ECG recordings revelead frequent and complex ventricular arrhythmias with dominant right bundle branch block morphology (Fig. 1).

A complete diagnostic work-up, including echocardiogram, stress test, ventriculography and coronary angiogram, failed to detect any organic substrate of the arrhythmia. No tachicardia was inducible by ventricular stimulation performed with an aggressive protocol. However the athlete was judged not eligible for competition due to the high weight of arrhytmic events and high heart rate of ventricular tachycardia (R-R cycle of less than 400 msec on ECG). In the follow-up period a cardiac MRI was performed showing normal left and right ventricular cavity dimensions and systolic function on cine images. At the level of the interventricular septum, pre- and post-contrast morphological images detected a focal area of fibrofatty replacement involving the inferior interventricular junction (Fig. 2A). The same MRI exam detected the presence of a vascular malformation consisting of right-sided aortic arch with atypical aortic arch configuration (incomplete aortic vascular ring) and the left subclavian artery arising from a dilated segment of the distal arch (Kommerell’s diverticulum) (Fig. 2 B).

**Fig 3.**
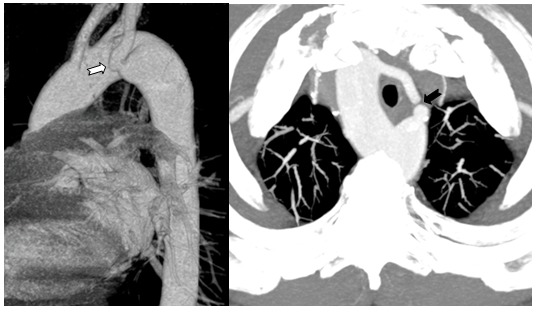
**Thoracic CT.** *On the left.* Volume rendering image for CT angiography of the thoracic aorta confirming that the left subclavian artery arises (white arrow) from a dilated segment of the distal arch (Kommerell’s diverticulum). *Right.* Maximum intensity projection (MIP) CT image of the upper thorax. Incomplete vascular ring for absence ligamentum arteriosum (black arrow). No signs of tracheal compression can be demonstrated.

Subsequently a thoracic CT scan demonstrated the absence of esophageal and/or tracheal compression, confirming the absence of a ligamentum arteriosum to complete the vascular ring (Fig. 3).

## Discussion

A left-dominant variant of arrhythmogenic cardiomyopathy (LDAC) has been recently described [6] and it is clinically characterized by the presence of arrhythmias from septal or inferolateral left ventricular myocardium in the absence of common pathological findings (i.e. ischemia, hypertrophy, heart failure). This condition has been linked to genetically determined defects of the desmosomes [7], the cell structures involved in the intercellular adhesion, similarly to the classic arrhythmogenic right ventricle cardiomyopathy with which also it shares the pathological and prognostic features. [8]

“Chronic” myocarditis is also a possible cause of apparently idiopathic arrythmia of the athletes. [9] Cardiac MRI is the gold standard for the study of ventricular volumes and function and the only tool for non-invasive tissue characterization. [10] It allows the identification of fatty infiltration and focal scar inside the myocardium. However, despite the valuable information provided from imaging techniques, diagnosis of certainty is only possible with genetic tests or endomyocardial biopsy. [11]

In the present case, detection on MRI of patchy myocardial fibrosis with subepicardial/midwall distribution, for lack of a medical history and of genetic or biopsy evidences, can not be attributed with certainty to the LDAC nor the myocarditis.

The Kommerell’s diverticulum [12] consists of the expansion of the origin of an aberrant subclavian artery, usually as part of a rare malformation of the aortic arch system. [13] It stems from an error of differentiation during embryonic development that involves the aortic arches. If the *ligamentus arteriosus* also persists a complete vascular ring can be generated, with consequent compression of the esophagus and trachea and need of corrective surgery. [14]

In our case cardiovascular MRI and thoracic CT, in addition to three-dimensional visualization of the heart and its relationship with the thoracic structures, have allowed the identification of abnormalities of the aorta that had escaped the cardiac examinations, even invasive, previously performed.

To our knowledge this is the first description of an association between left cardiomyopathy and Kommerell’s diverticulum. The etiological or pathophysiological link between the cardiovascular anomalies observed in this case appears unclear. However, the possibility that hemodynamic changes induced by vascular malformation might have favored the development of areas of myocardial tissue damage or predisposed to the the occurrence of myocarditis should not be excluded.

## Conclusion

The search for a structural heart disease is the primary task in the assessment of ventricular arrhythmia in athletes. However, the failure to detect a significant organic substrate in the initial stage of screening does not preclude the identification of structural pathologies in the follow-up by using advanced imaging techniques. In this way we were able to identify a unique combination of rare cardiovascular disease. Future observations and further investigations are required to elucidate their pathophysiologic link.

## Figures and Tables

**Fig. 2 A.**
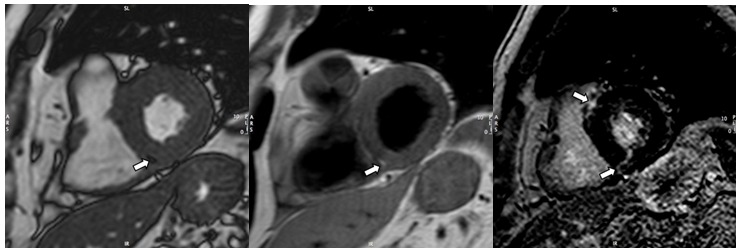
**Cardiac MRI.** *On the left*. Short-axis cine image: focal intramyocardial area showing “indian-ink sign” (arrow). *At the Center*. Fast-spin echo T1-weighted image obtained at the same level: area of signal hyperintesity (arrow) compatible with fatty infiltration. *Right*. Late post-gadolinium short-axis image with focal intramyocardial areas of hyperenhancement (arrows) involving the septal interventricular junctions (suggestive for myocardial fibrosis with non-ischemic pattern of distribution).

**Fig. 2B.**
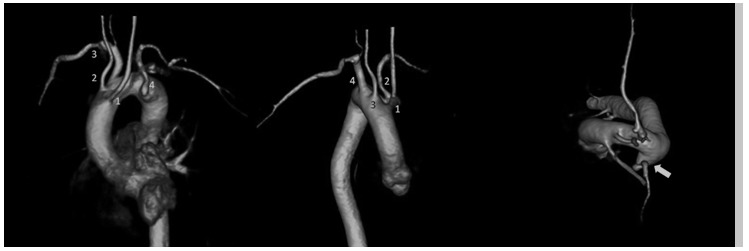
**MRI Angiography of the thoracic aorta** Volume rendering images. Anterior (*on the left)*, right (*at the center*) and superior (*right*) views:1: left common carotid artery; 2: right common carotid artery; 3: right subclavian artery; 4: left subclavian artery originating from the Kommerell’s diverticulum (arrow).
